# Computational Evaluation of B-Cell Clone Sizes in Bulk Populations

**DOI:** 10.3389/fimmu.2018.01472

**Published:** 2018-06-29

**Authors:** Aaron M. Rosenfeld, Wenzhao Meng, Dora Y. Chen, Bochao Zhang, Tomer Granot, Donna L. Farber, Uri Hershberg, Eline T. Luning Prak

**Affiliations:** ^1^School of Biomedical Engineering, Science and Health Systems, Drexel University, Philadelphia, PA, United States; ^2^Department of Pathology and Laboratory Medicine, Perelman School of Medicine, University of Pennsylvania, Philadelphia, PA, United States; ^3^Columbia Center for Translational Immunology, Columbia University, New York, NY, United States; ^4^Department of Microbiology and Immunology, Drexel College of Medicine, Drexel University, Philadelphia, PA, United States

**Keywords:** B cell, clone, antibody, immune repertoire, next generation sequencing, immunoglobulin, diversity

## Abstract

B cell clones expand and contract during adaptive immune responses and can persist or grow uncontrollably in lymphoproliferative disorders. One way to monitor and track B cell clones is to perform large-scale sampling of bulk cell populations, amplifying, and sequencing antibody gene rearrangements by next-generation sequencing (NGS). Here, we describe a series of computational approaches for estimating B cell clone size in NGS immune repertoire profiling data of antibody heavy chain gene rearrangements. We define three different measures of B cell clone size—copy numbers, instances, and unique sequences—and show how these measures can be used to rank clones, analyze their diversity, and study their distribution within and between individuals. We provide a detailed, step-by-step procedure for performing these analyses using two different data sets of spleen samples from human organ donors. In the first data set, 19 independently generated biological replicates from a single individual are analyzed for B cell clone size, diversity and sampling sufficiency for clonal overlap analysis. In the second data set, B cell clones are compared in eight different organ donors. We comment upon frequently encountered pitfalls and offer practical advice with alternative approaches. Overall, we provide a series of pragmatic analytical approaches and show how different clone size measures can be used to study the clonal landscape in bulk B cell immune repertoire profiling data.

## Introduction

The accurate measurement of clone size is fundamental to many immunological studies. B cells that are clonally related derive from a common progenitor cell. B cell clones can be viewed as the unit of selection in an immune response (1); the successful recruitment of clones results in diversification and expansion of cells with the appropriate antigen specificity and effector function (2, 3). Longitudinal studies of B cell responses, such as those tracking influenza-binding B cell clones (4, 5) require methods for measuring clone sizes and comparing them at different time points. Tracking B cell clones over time is also important for the diagnosis and monitoring of lymphoproliferative disorders such as chronic lymphocytic leukemia (6). Determining if a clone is likely to be present or absent in a population, as is the case for minimal residual disease testing (7), requires knowing or defining the analysis on the expected size of the clone and powering the analysis to detect clones of that size in the population (8, 9). Further complicating the analysis, the human B-cell repertoire contains a diverse collection of B cell clones of different sizes (9). Hence, clone tracking methods need to take several factors into account, including the level of sampling (the number of B cells being studied), the depth of sequencing (including the number of independently generated sequencing libraries per sample), and the distribution of clone sizes in the population being studied.

Here, we describe a series of computational procedures for estimating B-cell clone sizes in bulk populations using next-generation sequencing (NGS) data on antibody heavy chain gene rearrangements in genomic DNA (gDNA). The analysis of gDNA is the most parsimonious means of studying clone sizes on a large scale as each cell has only one template and many cells can be efficiently queried. Clonal overlap analysis and clone tracking typically require extensive sampling (10). Genomic DNA also provides information on non-productive gene rearrangements, providing a potential second target to identify a clone in B cells with two heavy chain gene rearrangements. Furthermore, DNA is less likely to be degraded than RNA, making it more versatile for suboptimal samples, such as those having low viability or those being derived from fixed tissues or cells. The analysis of the antibody heavy chain is most informative for clone identification and tracking because it has the most diverse CDR3 sequence (by virtue of the D gene segment, two rearrangement junctions and higher levels of non-templated additions and deletions at the junctions). IgH rearrangements amplified from gDNA are also the most often used sample type in the clinical setting, where parsimonious and robust assays are required.

With respect to the data generation, there are already several excellent protocols for immune repertoire profiling by NGS from DNA, RNA, and single cells (11–18). These different methods can be compared against one another on the same sample, along with procedures such as digital droplet PCR to perform experimental estimates on clone size (8). Single cell PCR methods, performed in emulsions or on beads provide a quantitative means of counting individual cells. These methods rely upon cDNA synthesis, either with reconstruction from RNAseq libraries or target capture-based approaches [reviewed in Ref (19)]. In addition to more straightforward quantitation (counting individual cells), single cell approaches can provide paired heavy and light chain IgH/IgL data from the same cell, providing additional fidelity for clonal assignment. One potential drawback of the single cell approach is that the efficiency of IgH/IgL amplification differs in different B cell subsets due to differences in RNA template abundance. The subset can be controlled by sorting or it may be possible to correct for these differences by measuring the recovery of IgH/IgL pairs from different subsets that are identified using other information about the cells (such as RNA transcript profiling within the same experiment). Of note, there have been recent advances in the generation of algorithms that deduce IgH/IgL rearrangements from single cell RNAseq data (20).

With bulk cell samples, one approach to measuring clone size experimentally is to use molecular calibrators (16, 21). With molecular calibrators, one or preferably several cloned standards are spiked into the reaction at known concentrations. For better quantification, multiple dilutions of standards are used, yielding a standard curve against which values of unknown rearrangements can be compared. In the log-linear range of the curve, quantification is most accurate. Standards can correct for differences in amplification efficiency of different VH or Vβ genes (21). One challenging aspect of molecular calibrators is that antibody genes can undergo somatic hypermutation (SHM). In fact, clones of interest often harbor somatic mutations when one is studying an immune response or certain forms of B cell neoplasia (such as follicular lymphoma or multiple myeloma) (22, 23). If mutations occur in the region of primer binding, the use of germline gene standards may not accurately model the PCR efficiency. For RNA-based libraries, a series of RNA spike-in standards has been developed that includes different murine and human VH genes, different lengths, different concentrations, and different levels of SHM (16, 24). The use of these standards following a protocol termed molecular amplification fingerprinting, allows for correction in PCR amplification efficiency and bias (16). While quite useful for understanding the nature of bias and error in the PCR amplification and sequencing steps (24), such calibrators have not yet been validated for broad use, particularly with pauci-cellular or suboptimal samples such as formalin-fixed paraffin-embedded specimens, where the calibrators may out-compete the lower quality sample templates.

Another method for evaluating clone size in bulk populations is limiting dilution analysis. In this method, one prepares serial dilutions of the sample and assays the rearrangements (or antibodies) of multiple replicates at different sample inputs (25, 26). The key to doing this well is to sequence several replicate libraries at each dilution factor. At limiting dilution, the event of interest is counted as present or absent and its frequency in the sample can be modeled using the Poisson distribution (27). As with single cell sequencing, this approach is expensive and requires extensive sampling and sequencing.

In our view, it is quite difficult to establish a “gold standard” for clone size estimation in bulk cell samples. Samples that contain mixed populations of cells with varying levels of SHM present a complex mixture of different templates for amplification. There can be PCR jackpot events, which can result in spurious clonal expansions. While many applications of clone tracking focus on large differences in clone size, with smaller clones or more subtle shifts in clone size, other factors come into play such as differences in sampling or library quality and sequencing depth. The advent of high-throughput sequencing has radically increased the number of cells we study when we analyze immune repertoires. Nonetheless, we still must assume in nearly all cases that our experiments are under-sampling the full diversity of the repertoires we are studying. To address this issue and ask questions about diversity and sampling of immune repertoires, we and others have turned to ecology for tools and methods (28–40).

**Figure 1:**
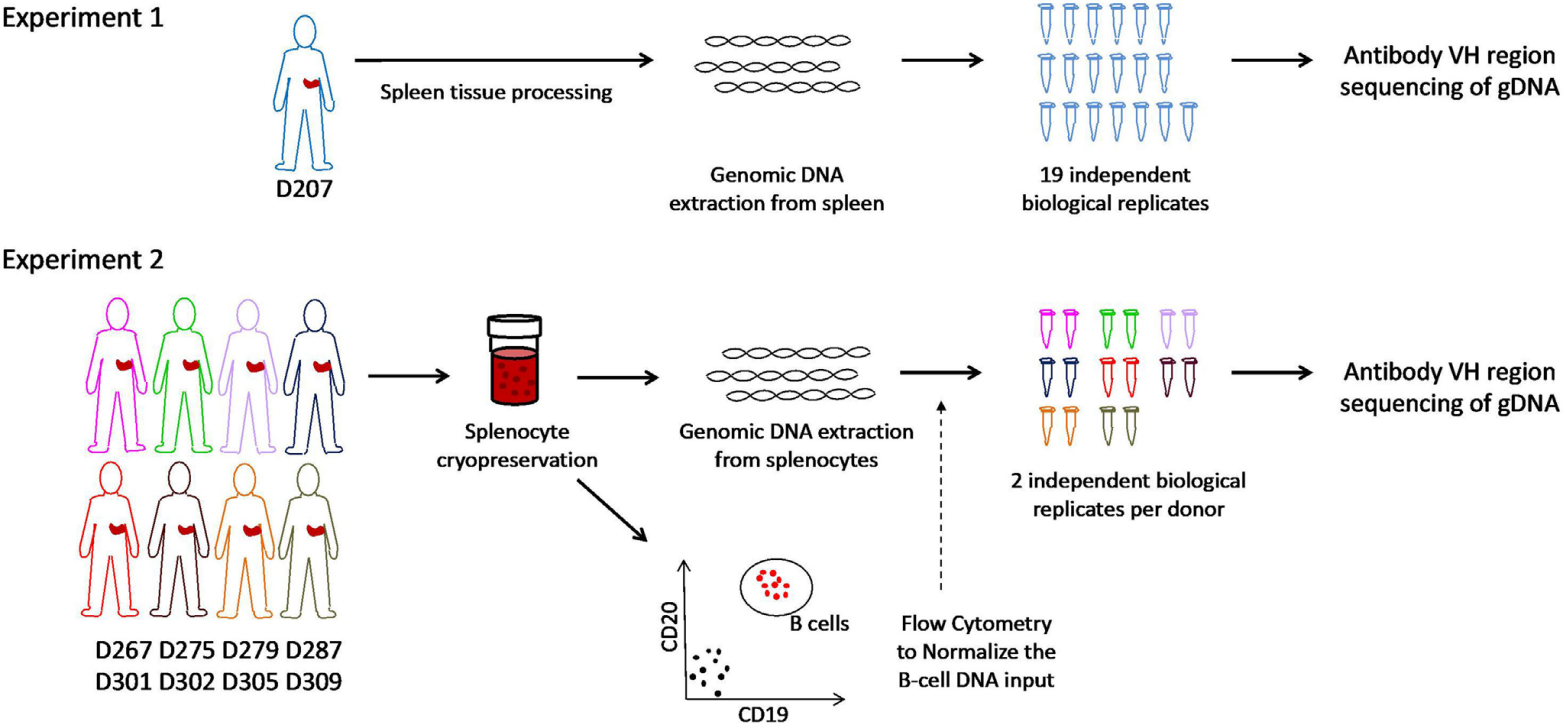
Schematic of experiments. In experiment 1, genomic DNA is extracted from spleen tissue of an organ donor. Immunoglobulin heavy chain gene rearrangements were amplified and sequenced from genomic DNA in 19 biological replicates. In experiment 2, splenocytes from eight different organ donors were cryopreserved at the time of organ recovery. All samples were thawed on the same day and analyzed for the B cell fraction by flow cytometry. Genomic DNA (normalized for the B cell fraction) was separately amplified and sequenced (two biological replicates per subject).

In this Protocol, we focus on sample-based computational procedures for evaluating clone sizes in bulk B cell antibody sequencing libraries. We describe and illustrate the use of metrics that rely on the analysis of individual sequencing libraries and repeating the analysis with multiple libraries per sample. We define three different metrics of B-cell clone size based upon sequence copies, instances, and unique sequences. To illustrate these procedures, we use two data sets from human spleen. We chose the spleen because it contains a complex mixture of B cell clones ranging in size (9). The spleen also contains abundant populations of memory B cells, providing a diverse mixture of B-cell clonal types, over a range of different SHM levels (41, 42). The spleen is also large, providing an ample supply of diverse clones for demonstrating clone size metrics that require different degrees of sampling. We describe our procedures using a large number of independently amplified sequencing libraries from the spleen of one organ donor and in a newly generated data set of spleen samples from eight different organ donors (Figure 1). Using these deep and survey-level sequencing data sets, we illustrate measures of within- and between-individual clonal size and diversity analysis. We propose a step-by-step approach that we hope will be useful for investigators who study clones in a variety of settings ranging from immune responses to malignancy.

## Materials and Equipment

### Donors

Human tissues used in this research were obtained from deceased organ donors through an approved research protocol and material transfer agreement with LiveOnNY, the organ procurement organization for the New York metropolitan area, as described previously (43). This type of research has been determined by IRBs of both the University of Pennsylvania and Columbia University to be non-human subjects research and, hence, ethics approval was not required, per institutional and national guidelines. A summary of donor information is provided in Table 1.

**Table 1:** Demographic characteristics of the organ donors.

Donor	Age	Sex	Race	Cause of death	WBC final	HCV	CMV	EBV
267	70	F	Black	CVA	11.3	0	1	1
275	31	M	White	Anoxia	17.8	0	0	1
279	73	F	White	CVA	25.4	0	0	1
287	34	M	White	Head trauma	5.6	0	1	1
301	33	F	Hispanic	Anoxia	29.8	0	1	1
302	56	M	Hispanic	Anoxia	16.1	0	1	1
305	28	F	White	Anoxia	9.0	0	0	0
309	45	F	Black	CVA	27.2	0	1	1
207	23	M	Hispanic	Head trauma	15.7	0	1	1

*Donor numbers are assigned by the Farber Lab. Age is in years. Cause of death is classified as cerebrovascular accident (CVA), head trauma, or anoxia. WBC, white blood cell count in thousands per microliter. Serologic status (IgG) for hepatitis C virus (HCV), cytomegalovirus (CMV), and Epstein–Barr Virus (EBV). 1 = positive; 0 = negative*.

### Sample Processing

Spleen samples were maintained in cold saline and brought to the laboratory at the University of Columbia within 4 h of organ procurement. Samples from D207 were processed as described [Experiment 1 in Figure 1 (9)] All other donor samples were rapidly processed to obtain lymphocyte populations, as described in detail (43, 44) and cryopreserved. Frozen cells were shipped on dry ice to the University of Pennsylvania. On the day of experiment 2 (see Figure 1), all of the cryopreserved samples were thawed and processed. Each sample was split into two aliquots. Genomic DNA was extracted from the first aliquot using a Qiagen Gentra Puregene cell kit following the manufacturer’s directions (Qiagen, Valencia, CA, USA, Cat. No. 158388). Flow cytometry was performed on the second cell aliquot to obtain the B-cell fraction. The following antibody-fluorophore combinations were used: FITC anti-CD19 (HIB19), PE anti-CD20 (2H7), APC anti-CD3 (HIT3a). Data were acquired on an LSRII flow cytometer (BD Biosciences, San Jose, CA, USA) and analyzed using FlowJo version 7.6.5 software (Treestar Inc., Ashland, OR, USA). The B cell fraction (CD19+CD20+CD3− divided by the total cells) for each donor spleen sample (except D207) is shown in Table 2.

**Table 2:** B cell percentages in spleen cell samples.

Donor	B cell (% total)	B cell (% ly gate)
267	8.48	33.76
275	12.2	18.27
279	25.4	39.76
287	37	43.65
301	6.32	13.67
302	15.6	28.12
305	35.6	54.17
309	7.11	28.6

*The B-cell (CD19+CD20+CD3− lymphocyte) percentage is calculated either out of the total mononuclear cells processed by Ficoll density gradient (total) or from the lymphocyte gate (ly gate). The % total was used to normalize the B-cell content between donors*.

### Antibody Heavy Chain Gene Rearrangement Amplification

The D207 sequencing libraries were generated as described previously (only the FR1 + JH amplified samples are included in this analysis) (9). For all samples from donors other than D207, sequencing libraries were amplified using a cocktail of VH1, VH2, VH3, VH4, VH5, and VH6 family specific primers in FR1 and one consensus JH region primer, adapted from the BIOMED2 primer series (45). Primers were synthesized by Integrated DNA Technologies (Coralville, IA, USA) and their sequences are provided in Table 3. The input DNA for amplification was normalized to the B cell fraction in the sample. For example, for D305, the B cell fraction (out of total cells) was 35%. To amplify 50 ng-equivalents of B cell gDNA from D305 spleen, we used 142.8 ng of input DNA (50 ng/0.35 = 142.8 ng). For each 25 µL amplification, primers were used at a concentration of 0.6 µM, gDNA normalized to represent 50 ng equivalents of B cell DNA, 0.2 mM dNTPs, and 1× PCR buffer with 1.5 mM MgCl_2_ using the Qiagen Multiplex PCR kit (Qiagen, Valencia, CA, USA; Cat. No. 206143) in molecular biology grade water (Millipore Sigma, St. Louis, MO, USA; Cat. No. W4502-1L). Amplification conditions for the PCR were primary denaturation at 95°C for 7 min, followed by cycling at 95°C 45 s, 60°C for 45 s, extension at 72°C for 90 s for 35 cycles, and a final extension step at 72°C for 10 min, using a Veriti 96-well thermal cycler (Life Technologies Corporation, Carlsbad, CA, USA) in 96-well plates (Denville, Holliston, MD, USA; Cat. No. C18080-10) sealed with Microseal B adhesive seal (BioRad, Cat. No. MSB1901). Amplicons were visualized on 1.5% agarose gels (Invitrogen/ThermoFisher, Waltham, MA, USA; Cat. No. 16500500) in TAE buffer, prepared fresh from 50× stock solution (Quality Biological, Gaithersburg, MD, USA; Cat. No. 351-008-491).

**Table 3:** PCR primers with Illumina adapters for human IgH rearrangement sequencing.

NexteraR2-Hu-VH1-FW1 5′-GTCTCGTGGGCTCGGAGATGTGTATAAGAGACAGGGCCTCAGTGAAGGTCTCCTGCAAG-3′
NexteraR2-Hu-VH2-FW1 5′-GTCTCGTGGGCTCGGAGATGTGTATAAGAGAC AGGTCTGGTCCTACGCTGGTGAAACCC-3′
NexteraR2-Hu-VH3-FW1 5′-GTCTCGTGGGCTCGGAGATGTGTATAAGAGACAGCTGGGGGGTCCCTGAGACTCTCCTG-3′
NexteraR2-Hu-VH4-FW1 5′-GTCTCGTGGGCTCGGAGATGTGTATAAGAGACAGCTTCGGAGACCCTGTCCCTCACCTG-3′
NexteraR2-Hu-VH5-FW1 5′-GTCTCGTGGGCTCGGAGATGTGTATAAGAGACAGCGGGGAGTCTCTGAAGATCTCCTGT-3′
NexteraR2-Hu-VH6-FW1 5′-GTCTCGTGGGCTCGGAGATGTGTATAAGAGACAGTCGCAGACCCTCTCACTCACCTGTG-3′
NexteraR1-Hu-JHmix1 5′-TCGTCGGCAGCGTCAGATGTGTATAAGAGACAGTACGTNCTTACCTGAGGAGACGGTGACC-3′
NexteraR1-Hu-JHmix2 5′-TCGTCGGCAGCGTCAGATGTGTATAAGAGACAGCTGCNCTTACCTGAGGAGACGGTGACC-3′
NexteraR1-Hu-JHmix3 5′-TCGTCGGCAGCGTCAGATGTGTATAAGAGACAGAGNCTTACCTGAGGAGACGGTGACC-3′

*While there is only one consensus JH primer, three primers with different spacers are used to generate sequencing diversity during sequencing from the JH end*.

### Library Preparation and Sequencing

Amplicons were purified using the Agencourt AMPure XP beads system (Beckman Coulter, Inc., Indianapolis, IN, USA; Cat. No. A63882) in a 1:1 ratio of beads to sample and eluted in 40 µL of TE (0.1 mM EDTA) buffer. 96-well plates with purified samples were sealed with adhesive aluminum sealing foil (RPI, Mount Prospect, IL, USA; Cat. No. 202502) and saved at −20°C if the second-round PCRs were not performed immediately following purification. Second-round PCRs (to generate the sequencing libraries with individual sample barcodes) were carried out using 4 µL of the first-round PCR product and 2.5 µL each of NexteraXT Index Primers S5XX and N7XX, using the Qiagen Multiplex PCR kit in a reaction volume of 25 µL. Amplification conditions for the library PCR were primary denaturation at 95°C for 10 min, followed by cycling at 95°C 30 s, 60°C 30 s, extension at 72°C 45 s for eight cycles, and a final extension step at 72°C for 10 min. Library amplicons were pooled and then subjected to two rounds of purification using the AMPure XP beads system. In both rounds of purification a 1:1 ratio of beads to sample was used, as before. After the first round of purification, the beads were eluted in TE buffer (1× Solution pH 8.0 with low EDTA, Affymetrix, Santa Clara, CA, USA; Cat. No. J75793-AP). Then, an equal volume of beads and TE eluate were mixed together and repurified. DNA concentrations of purified library preparations were measured using the Qubit 3.0 instrument (Invitrogen/ThermoFisher) with the Qubit dsDNA HS Assay Kit following the manufacturer’s instructions (Invitrogen, Cat. No. Q32851). Pooled libraries with a final concentration of 15 pM and PhiX control (titrated to be 10% of the concentration of the sequencing libraries; PhiX V3 Kit, Illumina Cat. No. FC-110-3001) were loaded onto an Illumina MiSeq instrument in the Human Immunology Core Facility at the University of Pennsylvania. 2 × 300 bp paired end kits were used (MiSeq Reagent Kit v3-600 cycle, Illumina, San Diego, CA, USA; Cat. No. 102-3003).

### Sequencing Run QC

Once the sequencing data are available, it is important to evaluate the quality of the sequencing run (46). We use the following metrics and cut-offs for run quality: (1) the percentage of clusters passing the Illumina sequencer instrument filter (%PF) is 90% or greater and (2) the percentage of sequences above the Phred quality score of Q30 (which is equivalent to the probability of an incorrect base call of 1 in 1,000) is 70% or greater.^1^ The use of paired sequences yields more information over a longer stretch of the V region than a single unpaired read. Paired reads also provide a consensus sequence at the termini of the reads, where the sequence quality tends to decline.

### Sequence Data Quality Filtering

Prior to using the ImmuneDB pipeline (31), pRESTO [described in Ref. (28)] was used for quality filtering of raw Illumina MiSeq sequences. First, each sequence was analyzed with a sliding window of 10 base pairs. If at any point, the average quality score within the window fell below 20, the sequence was trimmed from its beginning to the end of the window. To correct for single bases with low-quality, any base that had a quality score less than 20 was replaced with an “N,” indicating the uncertainty of the base call. Any sequence with more than 10 such N’s or a total length of less than 100 bases was discarded from the analysis. Code 1 shows the script used to run pRESTO with these parameters.

**Code 1:** Sequencing data quality control: The bash script used to run pRESTO.

FilterSeq.py trimqual -s *.fastq PairSeq.py \ --coord illumina \ -1 *R1*trimqual-pass.fastq \ -2 *R2*trimqual-pass.fastq AssemblePairs.py align \ --rc tail --coord illumina \ -1 *R1*pair-pass.fastq \ -2 *R2*pair-pass.fastq FilterSeq.py length -n 100 -s *assemble-pass.fastq FilterSeq.py maskqual -s *length-pass.fastq FilterSeq.py missing -s *maskqual-pass.fastq

### Alignment and Generation of Unique Sequences

After the QC steps described in Section “Sequence Data Quality Filtering,” each sequencing library has a set of high-quality sequences based upon the Phred quality scores. The next step was to use ImmuneDB (version 0.23.0) to determine the closest corresponding germline V- and J-gene for each sequence using an anchoring method (47). Once these V- and J-gene assignments were known, any sequence with less than 60% V-gene germline identity was discarded. Further, sequences were trimmed to IMGT position 150 to avoid primer biasing mutational analysis, and any sequence beginning after position 150 was removed.

ImmuneDB allows for multiple V- or J-genes to be assigned each sequence. This assignment can occur in two ways. First, if two germline gene sequences are equally and maximally similar to the input sequence, both will be assigned. Alternatively, if the maximally similar gene(s) is statistically indistinguishable from other genes, given the average mutation and length of sequences in the sample, all such genes will be assigned to the sequence. If any sequence has multiple V-gene annotations that are not from the same family, the sequence is discarded, as this likely indicates the sequence contains errors such as a hybrid PCR product. Sequences with cross-family J-gene annotations are not discarded; however, because many of these genes, especially human IGH J1, J4, and J5, are very similar to each other (47). At this stage of the process, the number of total reads and the fraction of valid reads are computed for each sequencing library (replicate). If the total number of valid reads (those containing identified V and J genes and passing quality, length, and primer trimming) from one replicate is very different (five or more times lower) than the other replicate, the sample in question is subjected to re-amplification and re-sequencing. The fraction of valid antibody heavy chain gene rearrangement sequences depends upon the stringency of filtering, but with the above-described parameters is typically 75–90%.

Once sequences are assigned V- and J-genes, the unique sequences are collapsed across the entire subject. Two sequences are considered the same if they differ only in positions where either sequence contains an N. This results in a set of sequences, which are unique within the subject, and have a corresponding copy number. The next step is to group sets of unique sequences, which likely share a common progenitor cell into clones. To prevent spurious clones from being constructed, sequences with a copy number <2 across the subject, those containing a stop codon in the CDR3, or those having any window of 30 nucleotides falling below 60% germline identity (indicating a potential uncorrected insertion/deletion) are excluded from clonal assignment. For the remaining sequences to be included in a common clone, they must share the same V-gene, J-gene, and CDR3 nucleotide length. Further, each pair of sequences within the clone must share at least 85% CDR3 amino-acid similarity by Hamming distance. The script to run the ImmuneDB pipeline with these parameters is shown in Code 2.

**Code 2:** ImmuneDB pipeline: The script used to run ImmuneDB. The germline files are included as supplemental files.

immunedb_admin create frontiers ~/configs immunedb_identify ~/configs/frontiers.json imgt_human_v.fasta \ imgt_human_j.fasta --trim-to 150 --max-padding 150 immunedb_collapse ~/configs/frontiers.json immunedb_clones ~/configs/frontiers.json similarity immunedb_clone_stats ~/configs/frontiers.json immunedb_sample_stats ~/configs/frontiers.json

### Tools for Immune Repertoire Visualization

The code for D20, cosine similarity, Hill number diversity plots, sample-based rarefaction curves, and clone metrics can be found at https://github.com/DrexelSystemsImmunologyLab/frontiers-clone-size-scripts. Resampling plots were created based on the method in Ref. (48) as implemented in https://github.com/bochaozhang/sampleRarefaction, which directly query ImmuneDB. For the clonal overlap analysis string plot, clones were exported from ImmuneDB using the immunedb_export command. Using this, clone tracking plots were generated using VDJtools requiring the CDR3 amino acids and V gene assignment to match. The command line for clone tracking in VDJtools is:

$$\begin{array}{l} \text{\$VDJTOOLS TrackClonotypes --i aaV \textbackslash{}}\\ \;[\text{sample}1\text{.txt sample}2\text{.txt sample}3\text{.txt}\ldots ]\text{ output\_prefix} \end{array}$$

Data were converted to Boolean values that, in turn, were used to generate string plots in CIMminer.^2^ For D20 and individual-based rarefaction analysis, VDJtools was used both to pool individual libraries (replicates were exported from ImmuneDB) and to generate the associated plots.

### Data and Method Sharing

Raw data and accompanying sample data are available on SRA under BioProject number PRJNA476510. In compliance with the Adaptive Immune Receptor Repertoire (AIRR) standard (49), steps for processing the raw data with pRESTO and ImmuneDB are available on Zenodo.^3^ Sequences annotated with ImmuneDB (those with VH gene and JH gene calls) are available *via* associated GenBank entries. All code to generate the database is provided in Code 2. Post-pipeline analysis scripts to generate data for plotting are available at (see https://github.com/DrexelSystemsImmunologyLab/frontiers-clone-size-scripts). Scripts used to generate plots are available upon request.

## Stepwise Procedures

Two data sets were generated for this analysis (see overview in Figure 1). In experiment 1, 19 independently amplified antibody heavy chain gene rearrangement libraries were generated from the spleen of D207. These libraries are part of a much larger data set on human organ donor tissues that was described previously. In experiment 2, survey-level sequencing of antibody heavy chain gene rearrangements was performed in eight additional organ donors in a newly created data set specifically for this study. In experiment 2, spleen samples from each donor were subjected to flow cytometry to determine the B cell fraction and antibody gene rearrangements were separately amplified in duplicate from the same number of input B cells in all eight donors. After generation of survey-level and deep antibody heavy chain gene rearrangement sequencing data and initial quality filtering, gene alignment and grouping of related sequences into clonal lineages (see “Materials and Methods”), we are ready to evaluate the clonal landscape, which is the focus of this Protocol.

### Different Metrics of Clone Size

**Figure 2:**
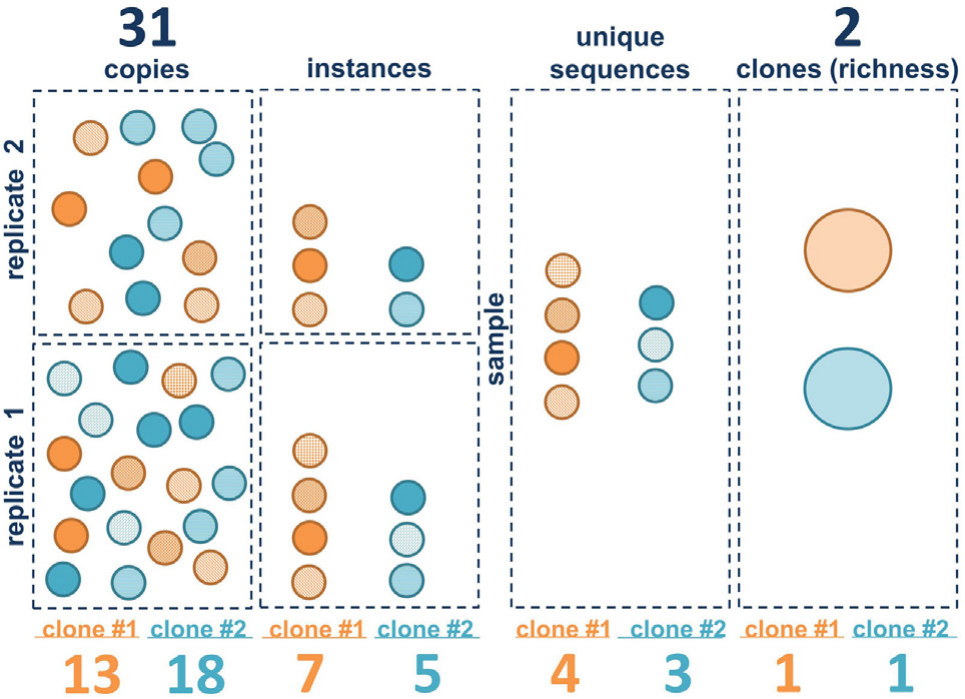
Clone size measure definitions. The first three vertical panels represent the three measures of clone size in this paper: copies, instances, and unique sequences. Each circle represents a B cell. Sequence variants within clone 1 (orange) and clone 2 (blue) are represented by different patterns in the circles. The right panel contains the clones (large ovals). Squares represent within the left two panels represent individual sequencing libraries whereas the entire collection of replicates from a single sample is shown in the right panels. Summed values (for both clones) for each metric are given above. Values for each individual clone are given below.

The clonal landscape of a B cell population can be viewed as a continuum of information content, ranging from maximal information with individual sequence copies to minimal information wherein each clone is only counted once. Borrowing terms from ecology, one can view each sequence copy as an individual and each clone as a species. The unique sequence variants within the clones generated by SHM are akin to quasi-species. We consider three different metrics of clone size in bulk population data: copies, instances, and unique sequences. Figure 2 illustrates these metrics for two hypothetical B cell clones in two separate sequencing libraries (replicates). The copies are like the individual B cells (although some B cells may have more than one sequence copy and some may have none, depending upon the depth of sequencing and on the level of sampling). Also, as discussed in the Section “Introduction,” it should be emphasized that extrapolating from copies to cells is challenging with bulk sequencing methods because there can be primer amplification bias. The next level down in information content is to ignore the copies and only count the number of times each unique sequence variant appears in each of the sequencing libraries. We call this measure instances. If the same sequence appears in both replicates, it is counted twice. If it appears in only one of the two libraries, it is only counted once. This measure is less sensitive to PCR amplification bias because the same bias has to occur with the same clone in independent replicates. But this measure is also affected by the depth of sequencing and the level of sequencing error, which can introduce spurious mutations that may be counted as unique sequence variants, depending upon how the data are filtered. The next level down is unique sequences. With unique sequences, all of the identical sequences from a single subject are grouped together and each unique sequence is only counted once. Here again, the measure can be influenced by sequencing depth and sequencing error. Finally, the most minimalist measure is to simply count the number of different clones, counting each clone only once, analogous to species richness. Richness does not capture information about clone size, only clone number.

It is important to emphasize that we are focusing here on measures of clonal size in bulk sequencing data. There are other metrics that could be used to estimate clone size, but they tend to be impractical in bulk libraries. For example, one could count instances of clones (rather than instances of unique sequences). Counting clone instances is a Boolean metric (presence or absence) wherein each clone is counted only once per sequencing library, if it is present. Thus, if there were two sequencing libraries, Boolean instances for all of the sampled clones would be either 1 or 2. A Boolean instances measure requires a very large number of sequencing libraries to be sensitive to differences in clone size (9). Such a metric is especially useful if single cell or digital droplet PCR is being used to measure clone size because, in those cases, hundreds or thousands of “libraries” can be queried.

### Initial Rearrangement Metadata Assessment

As an initial check of the sequencing data, we evaluate the numbers of copies, instances, unique sequences, and the number of clones (clones that are found in more than one sequencing library are only counted once). In Table 4, we present these data [obtained through the ImmuneDB pipeline (31)] in aggregate form for all eight donors at two libraries per donor and for one donor (D207) at 19 libraries.

**Table 4:** Sequencing data summary.

Subject	Libraries	Copies	Instances	Uniques	Clones
D207	19	5,526,691	1,921,080	1,895,669	136,876
D267	2	632,949	508,219	507,559	21,717
D275	2	446,178	340,315	339,787	15,161
D279	2	467,435	353,311	351,088	12,405
D287	2	537,427	360,998	360,967	9,111
D301	2	388,677	261,876	259,982	8,861
D302	2	504,337	380,141	379,360	14,333
D305	2	477,282	371,135	371,071	20,225
D309	2	412,672	361,225	360,807	17,186

*Each library was generated from separately amplified aliquots of DNA. Copies, instances, unique sequences, and clone numbers (see text) were compiled for all VDJ rearrangements (productive and non-productive) across all of the libraries from each donor using ImmuneDB. No copy number cut-off was used when computing the numbers of unique sequences or clones. The data from D207 have been published previously (9), but, here, we are only showing the data from that donor that were generated with the FR1 + JH primers, so only some of the libraries that were generated for the previous publication are shown here*.

We begin by comparing the estimated number of B cells to the number of unique sequences. Under conditions of maximal diversity [with each B cell in the population under study harboring at least one different heavy chain rearrangement, and assuming 100% yield and 1.4 VDJ rearrangements per cell on average because some cells will harbor more than one rearrangement (50)], we use the following formula to approximate the maximal number of B cell rearrangements per ng of input DNA:

(1) $$\text{max number of rearrangements}=\frac{\text{ng}\times 1000\text{ pg}/\text{ng}\times 1.4\text{ rearrangements}/\text{cell}}{6.7\text{ pg/cell}}$$

From a pure (flow cytometrically sorted) B-cell population, another useful estimator of the maximum number of cells (assuming 1.4 rearrangements/cell) is:

(2) 1 ng B cell DNA∼150 cells

**Figure 3:**
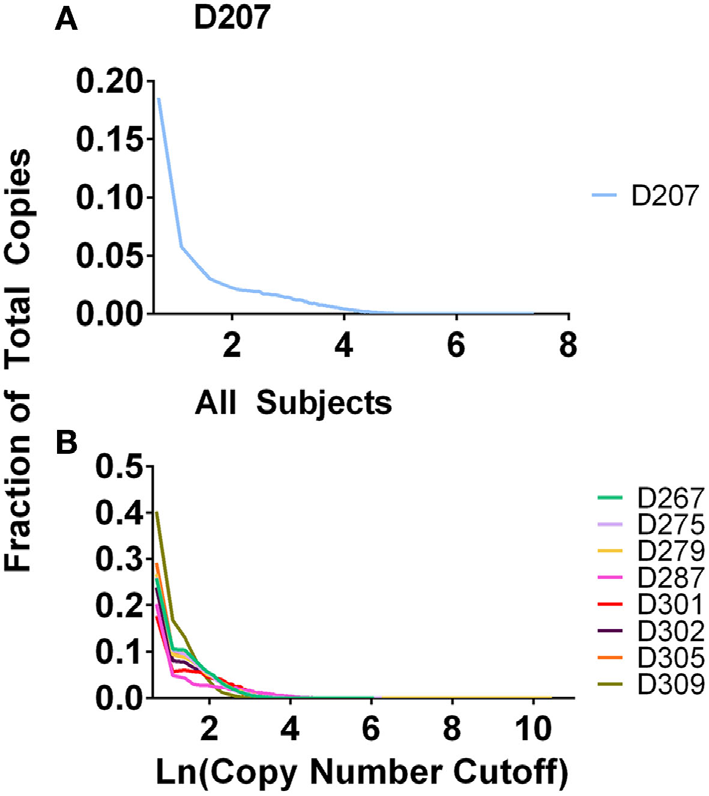
Fraction of total copies vs. clone copy number cut-off. **(A)** D207 (19 replicates); **(B)** all other subjects (survey-level sequencing).

In experiment 2, we accomplished normalization of the sample size by measuring the B-cell fraction using flow cytometry (Table 2) and then used the B-cell fraction to create the same number of B cell equivalents for each amplification. We used 50 ng of B-cell equivalent DNA in each replicate. Using the equations above, the maximum number of rearrangements that should be found in any one sample is 50 ng × 2 replicates × 1,000 pg/ng × 1.4 rearr/(6.7 pg/cell) = 20,895 unique rearrangements. All donors exceed this predicted maximum number of unique rearrangements. 20–40% of all of the sequences are present in one copy (Figure 3). Many of these sequences represent sequencing errors, whereas others represent infrequent clones. Because sequence copies are computed across all of the replicates, D207, with 19 replicates, has the lowest fraction of single copy sequences. In contrast to the excess of unique sequences, the number of clones is much closer to the theoretical maximum number of rearrangements.

### Reducing Sequencing Errors

To reduce the contribution of sequencing errors to clone size measures, one can employ several different strategies, often in combination (19, 46). The first is to use more stringent quality thresholds to filter the data. We can use quality scores of 30 or higher for bulk sequencing runs in which SHM is being analyzed. In addition, filtering of primer sequences, length trimming, and treatment of in/dels are important. The second approach to minimize sequencing error is to use a copy number filter. We typically use a minimum copy number of 2, which eliminates a lot of low copy sequences that are generated through sequencing error, but also eliminates valid low-copy sequences. One can rescue some of these valid low-copy sequences by computing clonal lineages across different replicates, which ImmuneDB can do (31). Thus, even if a sequence has a copy number of 1 in one library, if that same sequence is found in another library, it now has a copy number of at least 2 and can pass the filter. A third approach is to filter the data based upon instances or the number of times that the same unique sequence is found in different replicates. A fourth approach is to employ molecular barcoding (51). Molecular barcoding is typically performed on RNA samples and introduced *via* primers with variable sequence tags (“barcodes”) at the cDNA synthesis step [for a detailed method that can be applied to bulk populations, see Ref. (11)]. At sufficient sequencing depth, alignment of sequences with the same barcode is performed and used to generate a consensus sequence that is virtually free of sequencing errors. RNA-based assays tend to be lower throughput and require far more sequencing, increasing cost. Another approach to minimizing sequencing errors is to perform rolling circle amplification (52).

A final consideration involves processing of the sequencing data, which may affect how somatic mutations are identified and counted. For example, if the subject has one or more novel V gene alleles, rearrangements with these V genes may be scored as being mutated rather than matching the novel germline sequence. Software tools have been developed to search for novel alleles within individual samples, providing an individual reference database of germline and putative germline alleles against which sequences from the same individual are compared for mutation [see arXiv:1711.05843 (q-bio.PE), https://github.com/psathyrella/partis, and (53, 54)]. Filtering of low copy number sequence variants can eliminate valid sequences. To recover some of these sequences, one can construct algorithms that identify sequence variants that are shared (even among single copy sequences) in separate replicates from the same individual. ImmuneDB can do this handily because it takes all of the sequences from an individual into account when in constructs clonal lineages.

### Copy Number Cut-Offs and Clone Numbers

Table 5 shows the numbers of clones at different clone size thresholds (i.e., the clone size cut-off used in this illustration is instances) in each donor. As one would expect, the number of clones decreases as the threshold increases. One could envision setting the copy number threshold to be near a number that corresponds to the maximum number of unique rearrangements. An alternative approach is to discard clones at some fractional cut-off. For example, one could discard clones having sequences that fall below 50% of the mean copy number frequency of the sample. Either approach results in biases in the data. In the case of an absolute copy number cut-off, one runs the risk of discarding infrequent clones and the stringency of this cut-off will vary based upon the depth of sequencing: samples that are not as deeply sequenced (and have lower average copy numbers per template molecule) will lose more data than samples that are deeply sequenced. On the other hand, a relative copy number cut-off can be influenced by the copy number distribution of the sample. If a sample has very large clones in it, these large clones can skew the average copy number value and lead to excessively stringent filtering. Despite the fact that this experiment was controlled for differences in the B cell fraction, different numbers of clones were observed in different donors. Some of these differences appear to be due to intrinsic biological differences between subjects in their clonal landscape. Consistent with this idea, Table 5 also shows that the distribution of clones at different size cut-offs is not uniform across the different donors.

**Table 5:** Clone numbers with different instance cut-offs.

Subject	C1	C2	C3	C4	C5	C10
D207	136876	34441	12419	6,170	3,598	891
D267	21717	9,174	2,770	944	373	30
D275	15161	6,794	2,316	942	436	65
D279	12405	5,706	2,010	804	378	34
D287	9,111	5,910	3,669	2,047	1,195	98
D301	8,861	5,310	1,969	849	434	90
D302	14333	7,378	2,882	1,190	590	81
D305	20225	7,823	2,505	754	260	7
D309	17186	3,467	721	248	100	10

*The numbers of clones that were amplified with FR1 + JH primers are shown for different clone size cut-offs in each of the donors. The clone size cut-offs are given in instances (the minimum number of replicates that contain at least one sequence from the clone). C1 (at least one instance) is equivalent to no cut-off and contains all clones irrespective of size. C2 is two or more instances, C3 is three or more instances, and so on*.

### Visualization of Large Clones

A quick way of drilling down on the largest clones in a sample is to determine the fraction of the total copies that is comprised of the 20 highest copy number clones, also known as D20, which is defined in Eq. 3 where *c*_*i*_ is the copy count of clone *i*, and *T* is the total clone copy count.

(3) $$D20=\sum_{i=1}^{20}\frac{c_{i}}{T}$$

**Figure 4:**
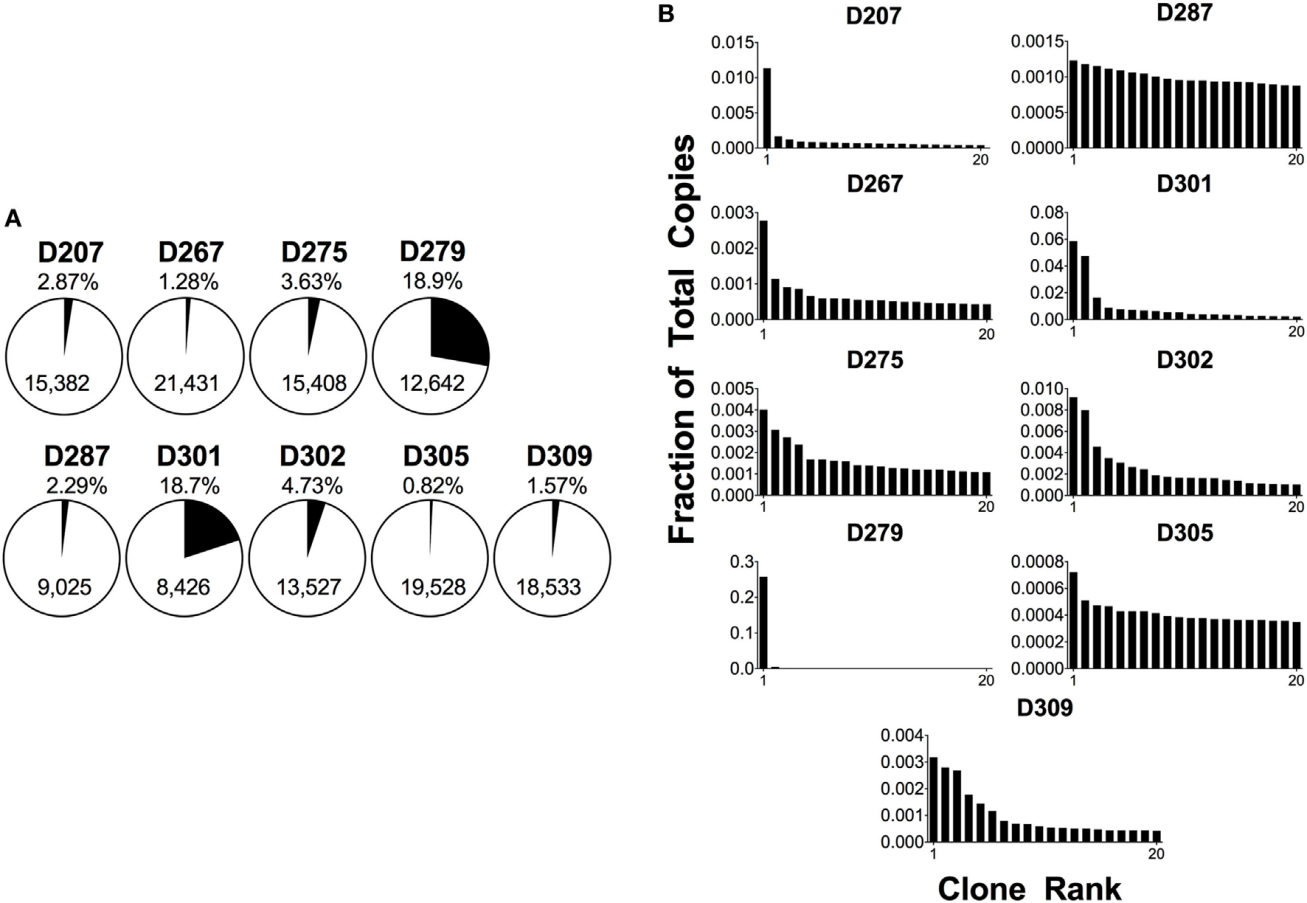
Analysis of 20 top-copy clones in different donors. **(A)** The 20 clones with the most copies in each subject are presented as a proportion of total copies in a given sample; the percentage is shown above the pie chart. The total number of clones per sample is in the body of the pie. **(B)** Histogram plots of the top 20 copy number rearrangements. The fraction of total copies for each rearrangement is plotted vs. the clone rank.

The D20 percentage can be over 90% in a patient with B-cell malignancy. Conversely, a blood sample from a healthy adult will tend to have many smaller clones and a corresponding D20 value of 1–2% or less. Figure 4A shows the D20 fraction and Figure 4B shows the copy number fraction of the 20 top copy rearrangements in survey-level sequencing from each of the nine organ donors. D279 and D301 have D20 values that exceed 18% of total copies. In the case of D279, a single rearrangement comprises over 15% of total copies. Furthermore, both of these donors have a rearrangement that is at least three times more frequent than the next most frequent rearrangement. In addition, two other donors, D207 and D287, each have top copy rearrangements that exceed the next most frequent rearrangement by more than threefold, but neither of these rearrangements exceeds 5% of total copies. The combined use of a frequency cut-off (such as 5%) and fold-change cut-off relative to the polyclonal background (such as threefold) provides greater confidence in declaring a true clonal expansion from oligoclonality. Furthermore, in both D279 and D301, there are several thousand B-cell clones, favoring clonal expansion over oligoclonality. At low B-cell numbers, PCR can be less efficient and jackpots (the disproportionate amplification of one dominant sequence) are more likely to occur (55). To further evaluate if these are bonafide clonal expansions, we measured the fractions of total copies in each individual replicate. In both cases, the fraction of total copies for each of the top two rearrangements was similar between the two replicates (the top copy rearrangement in D279 was 16% of total copies for replicate 1 and 17% for replicate 2; the top copy rearrangement in D301 was 5% in replicate 1 and 4% in replicate 2). The reproducibility of these values suggests that these rearrangements are present in one or two large expanded clones. If this analysis were being performed on peripheral blood samples, finding rearrangements of this size would be considered worrisome for pathologic clonal expansion, but we do not yet know the normal “reference range” of clone sizes in human tissues.

**Figure 5:**
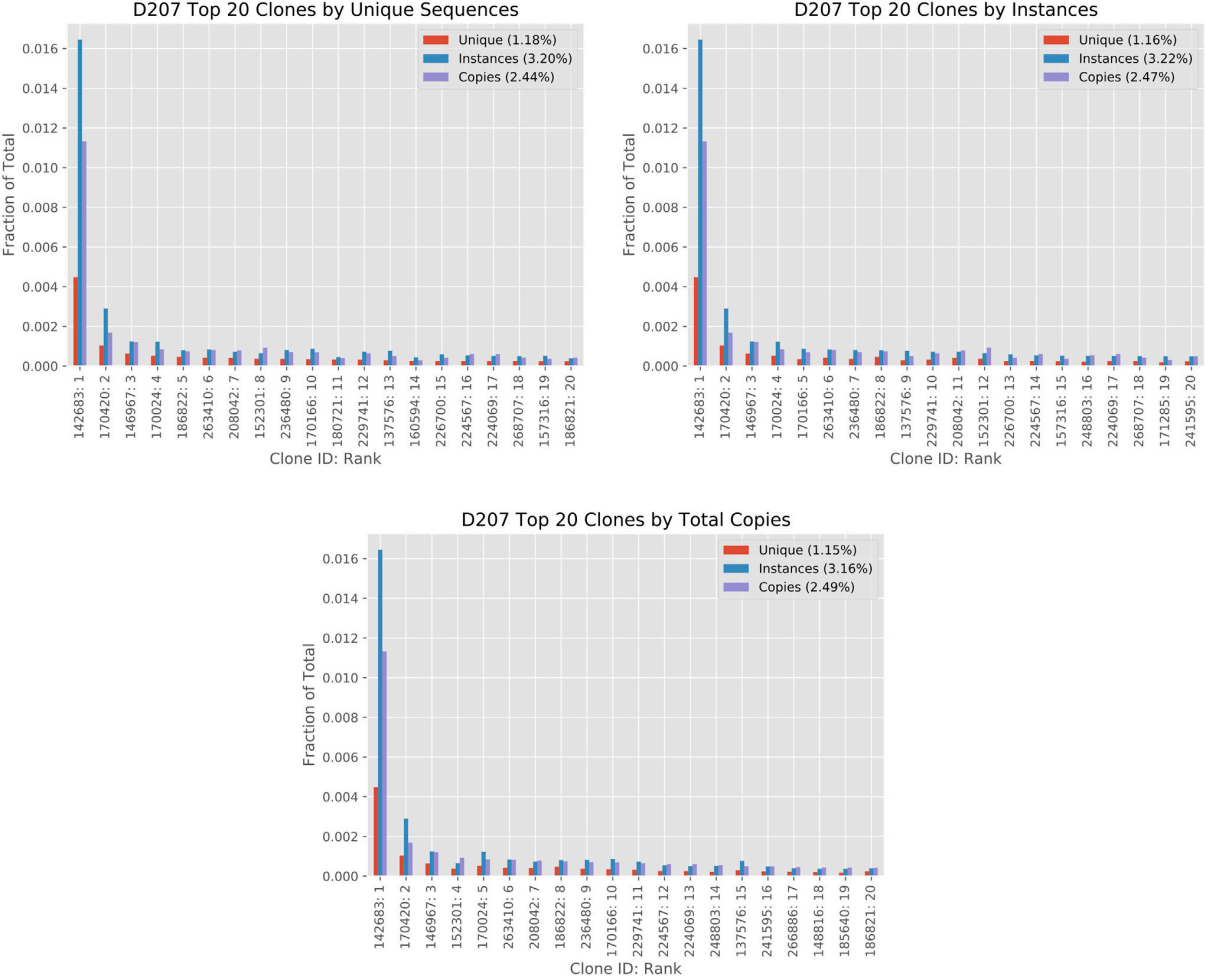
Analysis of top 20 clones by different clone size measures in D207. The top 20 clones are ordered in the three columns by total copies, instances, and unique sequences using the 19-library data set on D207. The *x*-axis shows the Clone ID (in ImmuneDB) followed by the rank. The clone size measures are given in fractions. The percentage within each chart legend indicates the sum of the top 20 ranked clones in the chart for each measure (please refer to the text for clone size definitions).

Figure 5 shows how different size measures compare for the top 20 ranked clones in D207. While many of the clones in the top 20 are found in all three ranking systems, their position in the ranking can shift and some clones are only found in a single rank. For example, clone #180721 (ranked eleventh by unique sequences) is not found in the top 20 clones ranked by instances or ranked by copies. One contributing factor to the difference in ranking is that instance and unique sequence-based measures have a much smaller dynamic range than copy number measures. A clone may have 10 times as many copy numbers but the same number of instances. There are far more “ties” in the instance-based measure. Biological differences may influence the rankings. For example, if there is a very large clone with minimal SHM, it may rank higher in a copy number-based rank than in a unique sequence-based rank.

### Diversity of Clones

In order to visualize the clonal landscape at different clone size ranges, one can plot the diversity of clones and give different weights to clones that are smaller or larger in size, as described in Ref. (39, 48). Here, the true diversity is given by:

(4) $${\;}^{q}D={\left(\sum_{i=1}^{R}p_{i}^{q}\right)}^{1/(1-q)}$$

The equation for, *^q^D*, true diversity at order (Hill number) *q*. *R* is richness, in this case the total number of clones, and *p_i_* is the proportional abundance of clone *i*. The abundance can be the proportional number of copies, unique sequences, or instances. In this equation, diversity is a unitless number that refers to the “effective” number of different clones in the population [see discussion in Ref. (39)]. Diversity is weighted by the parameter *q*. When *q* is 0, *D* is the number of different clones in the population. When *q* approaches 1, the diversity of each clone is proportional to its abundance (i.e., it is the weighted geometric mean). When *q* is greater than 1, larger clones are given more weight.

**Figure 6:**
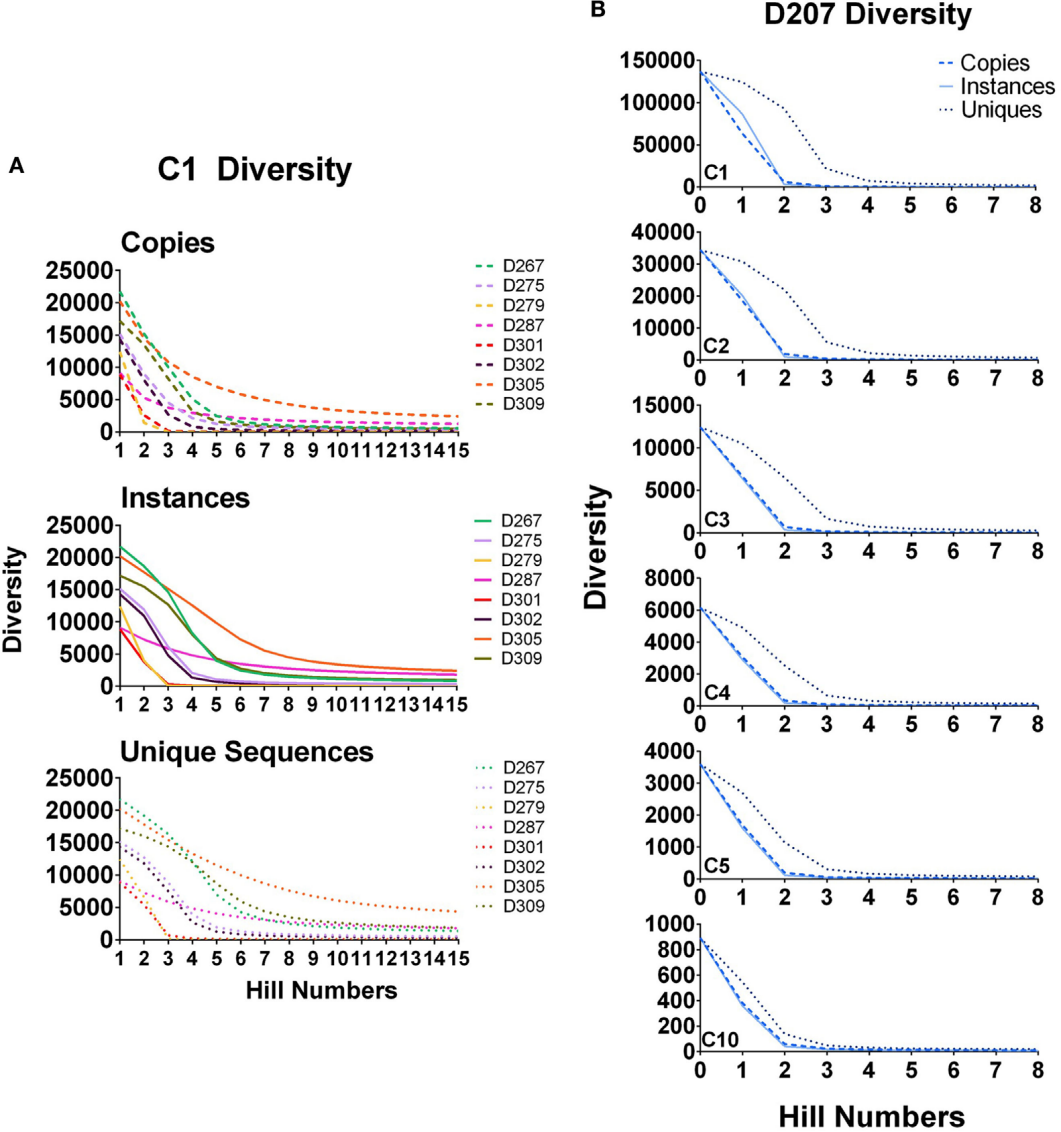
Diversity at different orders. **(A)** Diversity of C1 clones (all clones) calculated for all donors at survey level by instances, copies, and unique sequences; **(B)** Diversity of C1, C2, C3, C4, and C5 clones in D207. True diversity is calculated using copies, instances, and unique sequences (see text). Calculations are performed at different orders (Hill numbers). Higher orders give more weight to larger clones.

In Figure 6A, diversity is calculated for all clones (clones with 1 or more instances, marked C1) in all of the donors except 207. As the order increases, the diversity diminishes because there are far fewer large clones than there are small clones. Unlike the copy number cut-off plots, the diversity plots provide greater resolution of the representation of large clones in the population. At higher orders, D287 has a longer tail of medium to large-sized clones than the other donors. But among clones with at least five instances (C5 clones), D279 has the greatest diversity at higher orders.

In Figure 6B, diversity is calculated for clones of different size cut-offs in D207 (C1–C5 and C10 instances). Although we are using instances to filter the clones being considered, we then re-analyze the clone sizes of all of the clones meeting the instance cut-off using the three different size metrics: unique sequences, instances, and copies. Our test of sampling sufficiency is based on resampling and thus considers clone sizes in unique instances. However, at the clone size, we deem relevant when analyzing clonal diversity or overlap, there is still added information in considering different aspects of clone size. As stated above, both copy number and unique sequence number (and thus also instances) can be affected by PCR and sequencing artifacts. However, both also represent different indications of expansion—diversification by mutation for unique numbers and proliferation of specific types for copy numbers and instances. Thus, while they may not be totally faithful only to these dynamics, we do count and compare them, along with simply tracking the level of presence of clones in different samples.

### Descriptive Measures of Clonal Diversity and Evenness

**Figure 7:**
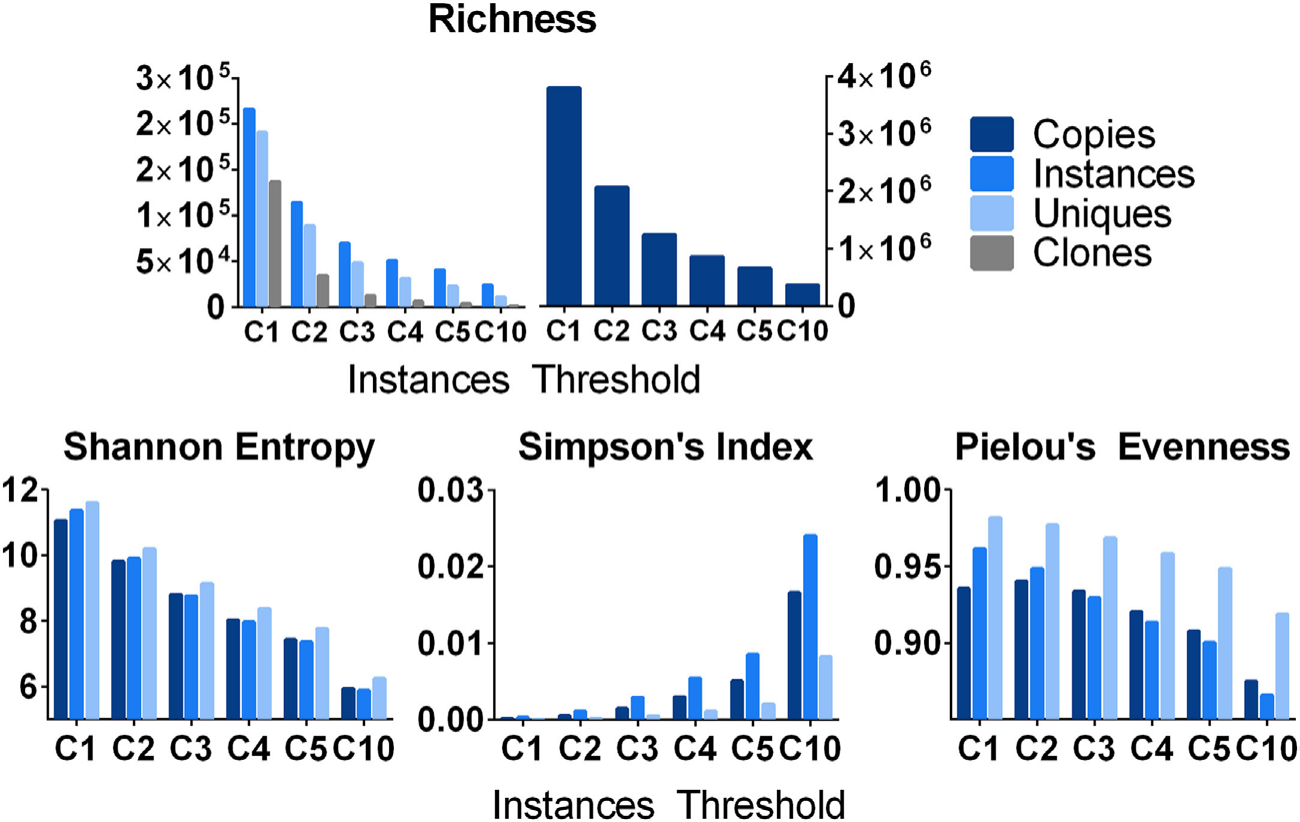
Various metrics of diversity and evenness. Count: the total number of clones and the number of instances, unique sequences, and copies that comprise them. Shannon Diversity: the Shannon diversity of clones when their size is defined as instances, unique sequences, and copies. Pielou’s Evenness: measures how evenly distributed clone sizes are. A value of 1 indicates all clones are of the same size, and a value of 0 indicates maximal diversity in size. It is defined as the Shannon Diversity divided by the maximal diversity [equal to ln(*S*)] where *S* is the number of clones. Simpson index: diversity at Hill order 2. This can be interpreted as the probability of randomly drawing two copies/instances/unique sequences that belong to the same clone.

Beyond the analysis of very large clones, one can study the distribution of clones within a sample using various descriptive measures of diversity or evenness or both (56). It is important to consider a sample may not be a single replicate, but multiple replicates from a common source. Shown in Figure 7 are analyses using 19 independent spleen replicates, which were analyzed from one subject (D207) and stratified by different clone size cut-offs based upon instances. The simplest metric is to count the number of species (a.k.a., richness, *R*) at each clone size cut-off using different clone metrics. Richness, which is equivalent to diversity of order 0, specifies the total number of species in a sample. As expected, richness decreases with increasing clone size cut-offs and decreases more rapidly for unique sequences and clones than for instances and total copies. Note that richness alone does not account for clone size; two samples with the same number of clones but drastically different clone sizes will still have the same richness. Therefore, it is useful to examine other metrics, which measure clonal size distribution in addition to species diversity. The Shannon entropy [*H* (57)] takes the number of individuals of each species (*i*), the proportion of sequences in a given clone over all of the different clones being measured (*p*), and the number of different clones (*R*) into account, as shown in Eq. 5:

(5) $$H=-\sum_{i=1}^{R}p_{i}\text{ ln }p_{i}$$

The Shannon entropy can be computed using copies, instances, or unique sequences as metrics for the numbers of individuals in each species (clone). For a given threshold (instance cut-off), all clones failing to meet the cut-off are filtered out and all of the remaining clones are used to compute *p_i_*. If nearly all of the sequences in a sample are found in one clone, the Shannon entropy approaches 0. Conversely, if all of the clones are equally abundant, the Shannon entropy approaches the natural logarithm of *R*. The Shannon entropy can also be computed with different logarithm bases.

Simpson’s index measures the true diversity (Eq. 4) at Hill order of 2. Simpson’s index measures the likelihood of encountering two sequences derived from the same clone when sequences are drawn at random from a given sequencing library or, in this case, a collection of 19 sequencing libraries from D207. It is defined by Eq. 6, where, as before, *R* is the richness and p_i_ is the proportional abundance of each clone.

(6) $$\lambda =\sum_{i=1}^{R}{p_{i}}^{2}$$

Counts, the Shannon entropy, and Simpson’s index are all influenced by sampling and by the depth of sequencing (which can cause spurious concentrations of sequences within individual clones in over-sequenced samples). Another frequently used metric, Clonality, takes on normalized diversity values ranging from 0 (maximally diverse) to 1 (monoclonal). Unlike entropy, clonality, *C*, measures the loss of diversity and can be represented as the inverse of entropy (*H*). One can also quantify how uniform clone sizes are using a measure of “evenness.” (58) Pielou’s Evenness is defined by Eq. 7:

(7) $$P=\sum_{i=1}^{R}\frac{p_{i}\text{ ln }p_{i}}{\text{ln }R}$$

Samples where most clones are of similar size will have an evenness measure closer to one whereas samples with predominant rearrangements will have a lower value. In D207, there are a few large clones, but the majority of clones are small, hence the overall evenness is very high. As smaller clones are excluded, the evenness decreases. When *R* is not known or if the clone size copy number cut-off is uncertain (resulting in variable inclusion/exclusion of low copy number clones across different sample types), this ratio can fluctuate and other ratio-based measures of evenness such as those described by Peet (59), may perform better (60).

Figure 7 shows that different metrics of richness and evenness (or hybrid measures of both) yield different results at different clone size cut-offs. Furthermore, when comparing results on different populations, the results from one measure do not necessarily translate intuitively to the results of another measure because the size distributions of clones in the different samples vary. For example, Figure 4 shows that D309 has a steep copy number cut-off curve at low copy number counts with a high proportion of low copy number clones, but also a shorter tail of higher copy number cut-off clones compared to most of the other donors. Conversely, D287 has fewer low copy clones, fewer intermediate size clones, but a longer tail of larger clones. For these reasons, we and others recommend analyzing the clonal landscape with several different metrics as well as plotting clonal diversity at different Hill numbers to visualize the clonal landscape in different clone size ranges (46). Some have also advocated using a collection of scalable and normalized diversity metrics to study immune repertoires (61).

### Rarefaction Analysis to Power Clone Size for Clone Tracking

**Figure 8:**
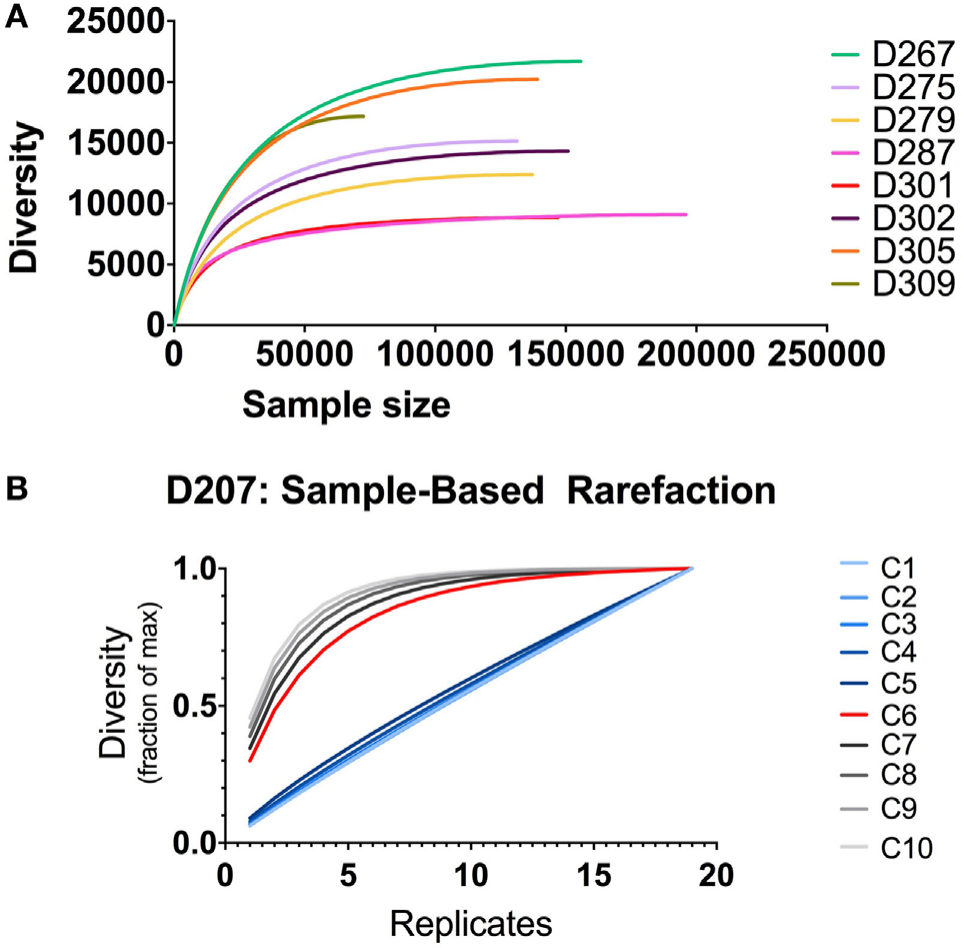
Rarefaction analysis. **(A)** Individual-based rarefaction with different sample sizes. Individual-based rarefaction plots were computed with VDJtools using unique sequences (no copy number cut-off) and subsampling to impute expected clone numbers. **(B)** Rarefaction analysis at different clone size cut-offs in D207. The expected number of clones in the population is given on the *y*-axis and the number of samples (replicates) is shown on the *x*-axis. Each line depicts rarefaction curves for clones with a given size cut-off. Clone size *C* is measured in instances (see text).

Tracking clones through different samples requires powering the analysis to detect clones of a given size. If one does not do this, then, the lack of clonal overlap between two samples could be due to insufficient sampling. The increment in finding new clones with additional sampling can be evaluated using rarefaction analysis (39, 40). Stratifying clones by size, one can generate rarefaction-based estimates of sampling adequacy, as illustrated in Figure 8. When there are only modest amounts of sequencing data (as we have here with only two replicates per donor), one typically relies on individual-based rather than sample-based rarefication analysis (Figure 8A). In this analysis, the diversity is the richness (number of different clones, analogous to the number of different species) and the sample size is the number of sequence copies (analogous to the number of sampled individuals). Different donors have different levels of diversity, despite the fact that we controlled for the B cell content. In all donors, the diversity is substantially lower than the number of sequences because several of the same or highly similar sequences comprise each clone. In some donors, such as D287, there many more sequences per clone than other donors (such as D267). One caveat to this analysis is that using individual-based rarefication can be unreliable when we count clone size by copy number: copy numbers may be inaccurate if sequencing depth and PCR amplification efficiency are not properly controlled.

Our most accurate indication of clone size and sequence abundance is the number of times we observe things in independently generated sequencing replicates. Beyond its ability to correct for sample-specific copy number inaccuracies, counting instances has the added advantage of being less influenced by samples that derive from a non-homogenous population (9). With more extensive sampling, we can perform sampling-based rarefaction analysis, as illustrated for D207 in Figure 8B. In this analysis, with larger clones (corresponding to size cut-offs of 6–10 instances), the curves level off. Rarefaction analysis, coupled with clone size, can be used for power detection of clonal overlap or clone tracking between samples. In this example, clones with an instance cut-off of 6 (C6, red curve) are the optimal size for overlap analysis in this data set: they are the smallest size clone with a rarefaction curve that levels off. As one would expect, there is a trade-off between the amount of sampling required and the likelihood of capturing a clone. However, when two populations have very large numbers of overlapping clones or very large clones, a lower capture threshold may be sufficient to adequately sample clones of interest.

### Clonal Overlap Analysis

To determine if two samples contain overlapping clones, the most straightforward thing to do is to count the number of clones that overlap and compare that number to the total number of clones in each of the samples. This type of counting is the basis for generating a Venn diagram. However, such Venn diagrams are hard to compare quantitatively as they do not take different sizes of clones into account and they become visually cumbersome when more than two samples are being compared. In lieu of this approach, several metrics have been developed to quantify overlap, giving weight to clone sizes in the samples, including the relative overlap diversity, the geometric mean of relative overlap frequencies, and the clonotype-wise sum of the geometric mean frequencies. This metric is easy to calculate and is not overly influenced by the relative sizes of clones and, as it ranges from 0 to 1, it is easy to compare across experiments. Here, we illustrate the use of the cosine similarity metric for quantifying overlap between two-sample pairs. The equation for the cosine similarity metric is:

(8) $$\mathrm{cosine similarity}=\frac{\sum_{i=1}^{n}A_{i}B_{i}}{\sqrt{\sum_{i=1}^{n}A_{i}^{2}}\sqrt{\sum_{i=1}^{n}B_{i}^{2}}}$$

The cosine similarity is computed for two samples *A* and *B*, for example, replicate 1 from D207 and replicate 2 from D207. Both *A* and *B* are vectors of length *R*, where *R* is the number of unique clones across the two samples. The value of *A_i_* or *B_i_* is the abundance of clone *i* in sample *A* or *B*. The abundance can be any of the measures of clone size such as copies, unique sequences, or instances.

The cosine metric is useful for evaluation of clonal overlap between two samples, but does not provide a means of comparing overlap of clones that span multiple samples.

**Figure 9:**
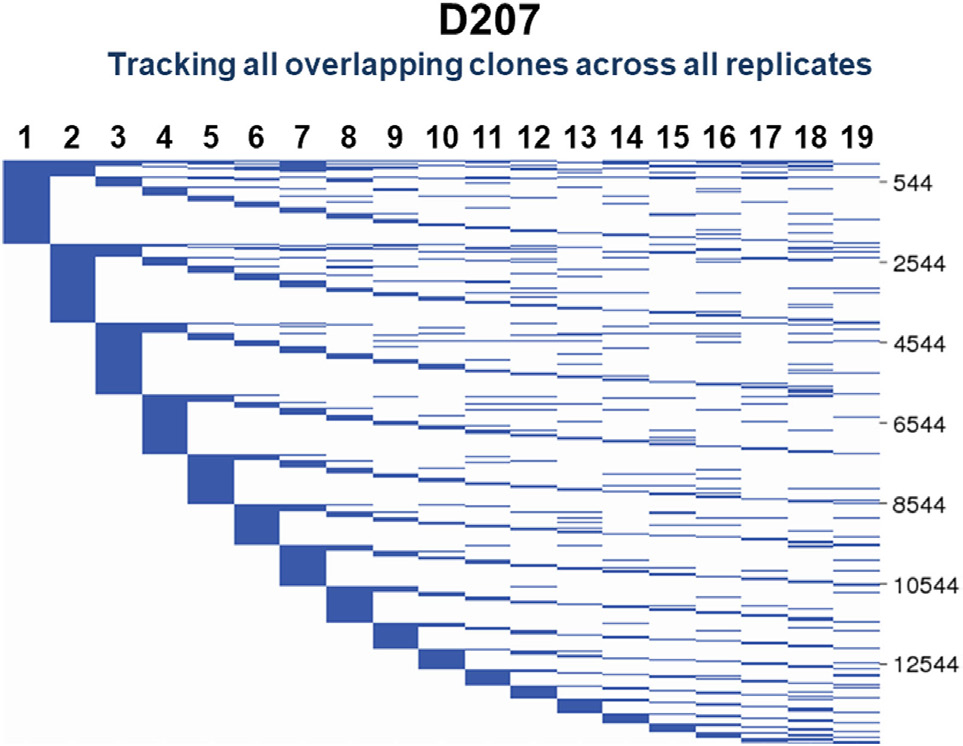
Tracking of shared clones across replicates in D207. All clones that were found in two or more replicates are shown. Each column is a replicate. Each horizontal line is a clone. The numbers of shared clones are given on the scale to the right.

To visualize clones found in three or more samples, we use string plots. String plots based on Boolean values (presence or absence of a clone in a replicate), are shown in Figure 9 for all clones (*n* = 14,543) that overlap in at least two replicates in D207. In these plots, the strings (horizontal lines) represent the individual overlapping clones. The overlapping clones comprise 11% of all C1 clones in the 19 libraries of D207 (overlapping clones have been removed from this total C1 clone number). Note that over 90% of these overlapping clones in the entire data set have already been discovered in the first 10 sampled replicates. In these plots, the strings can also be colored based upon a metric of clone size such as percentage of copies within a sequencing library (34).

One can add other dimensions to string plots, as we did with “line circle plots” in our analysis of clonal representation in different tissues (9). In line circle plots, each line represented a clone, circles indicated membership of that clone in a tissue, the size of the circle indicated the number of copies of the clone in the tissue samples, and colored wedges in the circle indicated what fraction of sequencing libraries from that tissue contained members of the clone. Thus, in line circle plots, one can display three different features of a clone—its tissue membership, its copy number in the different tissues, and its instance number—within replicate libraries from each tissue. The plots can of course be modified to display different parameters, depending upon the comparisons of interest. String plots provide a means of visualizing overlapping clones, but it is important to remember that they focus exclusively on the overlapping clones. The appearance of a string plot can be misleading if the samples that are included in the plot are of unequal size or clonal composition. The total number of observed clones within each sample being compared needs to be considered to determine if the numbers of overlapping clones reflect meaningful overlap or merely sampling differences. To visualize all of the clones in the samples being compared, Venn diagrams can be used.

## Anticipated Results (Pitfalls, Artifacts, and Troubleshooting)

### Overview of Clone Size Evaluation

Here, we present a series of analytical approaches to evaluate B-cell clone size in bulk populations. We describe three basic types of immune repertoire measures that are impacted by clone size. The first is the fraction of total copies that harbor the one or two-most frequent rearrangements. This fraction is typically higher than 0.05 for a malignant clone with a polyclonal background and may require an even higher cut-off with an oligoclonal background. We also determine the fold increase of the most frequent rearrangement compared to the next most frequent rearrangement in the sample. Ideally, this change should be threefold or greater. These criteria tend to be sufficient for relative or approximate measures of clone size. For smaller clones or finer resolution of clone size, calibration to an external standard (such as cloned rearrangements that are spiked into the sequencing reaction), single cell counting, or limiting dilution analysis may be required. We perform sequencing reactions in at least duplicate to rule out spurious clonal expansions due to oligoclonality (accompanied by poor reproducibility in the replicates) or PCR jackpots.

The second measure is clonal diversity, which can provide insights into immune competence or robustness of a targeted immune response, such as tumor infiltrating lymphocytes in a biopsy specimen. At the bulk population level, clonal diversity needs to be visualized at different orders (Hill numbers) to give weight to clones of different size in the population. If different samples are to be compared, it is important to normalize the input DNA of the sequencing library for the B cell content in different samples. This can be accomplished by sorting cells with a specific phenotype or by performing FACS analysis and determining the B cell fraction in the cell suspension, as we did here. If we had not controlled for the B cell content, then differences in diversity could have reflected differences in sampling rather than true differences in diversity. A second important consideration with diversity analysis is to visualize the clonal landscape at different clone sizes. We describe two ways to accomplish this: first, plot the number of clones at different clone size cut-offs, and second, plot the true diversity at different orders, giving different weight to clones of different size ranges. Two individuals may have very similar small clones, but one person may have many intermediate-size clones while another may have a few really large clones. If one only visualized the data with a single diversity measure, one might miss features of the clonal landscape that distinguish one sample from another.

The third measure is clone tracking, including clonal overlap analysis. The ability to track a clone depends upon its frequency in the population; thus, the respective sizes of the clone and the population in which the clone resides both matter. Starting with sequences from bulk populations, we recommend using sample-based rarefaction analysis to determine the clone size that can be detected reliably and the number of sequencing replicates needed to adequately sample the clone. The null hypothesis of this analysis is that the clone size is the same in the two populations being sampled and evaluated for clonal overlap, which often is not the case. We recommend tailoring the clone size metric to the types of clones being compared. For clones that harbor substantial somatic mutations, the number of unique sequence variants per clone may be a useful clone size metric. On the other hand, if samples differ in their sequencing depths, it may be useful to deploy an instance or even a Boolean (presence vs. absence) metric for clone size, although such metrics typically require very extensive sampling and may be impractical. Finally, if amplification efficiency, sequencing depth, and B cell content are well controlled, it may be possible to use copy number fractions. To visualize clonal overlap, we recommend using the cosine statistic for two-sample comparisons and string plots for three or more sample clone tracking experiments.

### Real-Life Limitations and Alternative Approaches

Samples may be limited in quantity or quality, or we may not know the B-cell content. Small and low-quality samples can be encountered in fixed tissue samples. Due to poor DNA quality, it may only be possible to generate short amplicons from such samples, potentially reducing the fidelity of V gene assignment (47). Furthermore, modest numbers and/or fractions of B cells in such samples can increase the likelihood of PCR jackpots and the accumulation of sequencing errors due to over-sequencing of the few templates that are present. Clone size measurements in such samples may not be reliable or even possible.

To judge the adequacy of the library, we use the quality metrics described in Sections “Sequencing Run QC” and “Sequence Data Quality” Filtering and look at the number of clones. If the sample has fewer than 50 clones and/or 1,000 valid sequences, it may be challenging to identify a dominant clone unless the sample is nearly monoclonal. Replicate amplifications from oligoclonal samples lacking clones will tend to reveal different clones in the replicate, whereas samples with clonal expansions will reveal consistent amplification of the same clones if they are large enough. Sometimes it is not possible to know the B-cell content in a sample. If the B-cell content is unknown, we calculate the copy number distribution and average copy number, and then use a fraction of the average copy number as the copy number cut-off. For example, if the average copy number is 100, the copy number cut-off might be as high as 10 or 20, whereas if the average copy number is 2, there might be no cut-off or only sequences with a copy number of 1 may be eliminated.

### Community Efforts to Validate and Standardize Repertoire Analysis Tools

All of the clonal size measurements introduced for studying bulk populations of B cells assume that the annotation of clones (and, therefore, genes) is correct. There are many tools that claim to achieve this, including ImmuneDB, which was used for this paper. However, there has been no robust method for determining how well the clonal associations produced approximate the true clonal landscape. It may be possible to validate tools under specific conditions when the clones are known *a priori*, but currently there is no universal standard by which tools can be tested.

There are at present several hurdles to creating such an estimate, some of which may be insurmountable. We start our germline association of sequences from an expanded population whose somatic and germline history is ill-defined. To estimate the accuracy of our association, we would need to know which germline genes, and what copy numbers are present in the subject and what kinds of selection pressures have created the gene segment usage in the active repertoire. This final requirement is quite difficult, as selection has been shown to skew repertoires significantly and in a very individual way (62). In addition, there are differences that simply cannot be detected after the fact, such as discrimination between germline genes that are too similar to tell apart (47).

There is a goal of generating such a standard within the AIRR Community, with active discussion in the B-T.cr forum.^4^ An alternative method is to generate sequences *in silico* with known V- and J-gene assignments (63), run a gene inference tool on the sequences, and see how well the results match the input. However, this approach assumes that we can write software that adequately mimics the underlying biological processes.

### Use of Multiple Tools on the Same Data Set

One approach to validation of an analysis method, when lacking a “gold standard” for comparison, is to use multiple clonal assignment tools and compare the results. Even though the results will likely not be identical, at least for the large clones, one would expect similar clonal assignments to be produced. The size analyses from this paper could then be applied at the threshold of clone size to which the tools generally agree.

Starting with the sequencing data table, we begin with a back-of-the-envelope equation on the maximum predicted number of gene rearrangements. If the number of unique sequences in the sample exceeds this value, we look at other quality metrics to determine if there is adequate filtering of the data to remove sequencing errors. If the number of unique sequences in the sample is 10-fold or more below the maximum predicted number of rearrangements (Eq. 1), we review the experiment to determine if there is any explanation for the low recovery of rearrangements, such as a low-quality sample or perhaps an unexpectedly low B cell fraction. We also look more closely at the data filtering to see if we are discarding too much data in the filtering process.

While beyond the scope of this paper, the method for grouping related sequences into clonal lineages could also influence downstream measurements of clonal diversity, size, and overlap. Several different approaches for associating antibody sequences into clonal lineages exist (29, 32, 35, 38, 64). Different methods as well as different parameters within individual methods can be tested to determine if the findings are robust. These methods make different assumptions and can result in different stringencies of clonal association under different conditions [such as different levels of SHM, discussed further in Ref. (9)]. We often try processing data with two or more different pipelines, such as MiXCR (34) and ImmuneDB (31). Getting similar answers with both pipelines encourages us that the result is more likely to be robust. When there are discrepancies, they can be due to differences in sequence quality filtering or the clonal lineage assignment steps. In ImmuneDB, one can compare the total number of sequences to the number of “identifiable” sequences, which are those to which a V and a J gene (or gene tie) have been assigned. If there is a massive loss of sequences going from total to valid sequences, the pipeline may be filtering out sequences of interest or there may be poor sequence quality. Another quick sanity check is to look at the fraction of sequences with productive rearrangements. In mature B cells, a low fraction of productive rearrangements (<75%) is usually an indicator of poor sequence quality.

For clone size analysis, if only clones with at least six instances (C6) are inferred similarly with multiple tools, it may be possible to limit the analysis to clones of size C6 and above. However, it may not always be desirable to focus only on a small set of large clones, as the discarded clones typically comprise the vast majority of the sampled repertoire. Additionally, different tools make different basic assumptions, sometimes making comparisons difficult. There is also the question of how stringent one needs to be to declare two clonal assignments “similar.” Finally, in some cases, differences observed with different tools are not due to problems with the data or the analysis but rather are due to bonafide differences in what the analytical tools are actually measuring in the data set. For example, comparing diversity at different orders can result in different answers because they give different weights to clones of different sizes. In the end, the best course of action is to pick the metric that best captures the clones of interest in the population.

### Replication

As with most experiments, one of the most reliable methods for determining if results are valid is to replicate them by making additional measurements or by performing additional experiments. In the data we present here, we illustrate two forms of replication. The first is that we perform the same bulk sequencing analysis on nine different organ donors. This analysis reveals certain features that are shared in all donors (such as the preponderance of small clones having 1–2 sequences per clone) and other features that vary between different donors (such as the proportion of very large clones, D20, or the distribution of clone sizes). If we had only analyzed two donors (such as D267 and D309), we might have concluded the different individuals have rather similar clone size distributions in the spleen.

Replication is also achieved by making additional measurements on the same sample, as we showed here with 19 replicates from D207 spleen. With antibody gene rearrangement sequencing from gDNA, performing additional amplifications and sequencing on the same DNA aliquot is analogous to sampling additional cells from the same cell population since each cell only has one template molecule. As we show here and in Ref. (9), for clone tracking studies, it is important to power the analysis on both the clone size and the degree of sampling.

## Concluding Remarks

In this paper, we demonstrate the importance of choosing the appropriate combination of experimental approaches and analytical tools to measure B-cell clone size. One has to know what scale of clone sizes is of interest, which means visualizing the repertoire as a whole on a diversity or clone copy number cut-off plot. Additional considerations that guide the choice of clone size metric include the prevalence of SHMs, the possibility of uncontrolled differences in sequencing depth, and the availability of replicate libraries. For clonal overlap analysis, there are approaches that quantify the degree of overlap and others that focus on the similarity of the overlapping clones themselves. Finally, analysis tools and experimental approaches, especially in the single cell realm, are undergoing rapid evolution (19, 20). Members of the AIRR and RepSeq communities, including many of the research teams that have contributed to this special research topic in Frontiers, are contributing to experimental approaches, data analysis and data sharing as methods and providing recommendations (49, 65). We look forward to a future for clonal analysis that is filled with promise and complexity.

## Ethics Statement

Human tissues used in this research were obtained from deceased organ donors through an approved research protocol and material transfer agreement with LiveOnNY, the organ procurement organization for the New York metropolitan area. This type of research has been determined by IRBs of both the University of Pennsylvania and Columbia University as non-human subjects research and, hence, an ethics approval was not required as per institutional and national guidelines.

## Author Contributions

EP, UH, AR, and WM contributed to the conception and design of the study; DF directs the organ donor tissue resource for acquisition of tissue samples; TG assisted with organ donor sample processing, shipping, and provided input into experimental design; WM generated the flow cytometry and sequencing data and contributed to the data analysis; AR organized the database and performed the clonal rank, rarefaction, and diversity analyses; BZ developed the software to measure resampling; DC generated the clone size cut-off and individual rarefaction plots and designed all of the figures. EP wrote the first draft of the manuscript and oversaw the overall study; UH, AR, WM, and DC wrote sections of the manuscript. All authors contributed to manuscript revision, read and approved the submitted version.

## Conflict of Interest Statement

The authors declare that the research was conducted in the absence of any commercial or financial relationships that could be construed as a potential conflict of interest.

## Footnotes

^1^https://www.illumina.com/documents/products/technotes/technote_Q-Scores.pdf (Accessed: November, 2017).

^2^https://discover.nci.nih.gov/cimminer/oneMatrix.do (Accessed: November, 2017).

^3^DOI: 10.5281/zenodo.1292010.2017.

^4^https://b-t.cr/t/how-to-decide-on-an-example-data-set-to-use-for-testing-software/129 (Accessed: November, 2017).

## Supplementary Material

Table S1 Fasta files of IMGT human JH genes used for germline gene assignment.

Table S2 Fasta files of IMGT human VH genes used for germline gene assignment.
